# Obesity significantly elevates thromboembolism risk in severe trauma, yet essential resuscitation with TXA and erythrocytes remains safe

**DOI:** 10.1007/s00068-025-03061-9

**Published:** 2026-01-13

**Authors:** B. Erdle, Marie Knoll, F. C. Wagner, A. Frodl, T. D. Diallo, J. Kalbhenn, H. Schmal, Nils Mühlenfeld

**Affiliations:** 1 Department of Orthopedics and Trauma Surgery, Faculty of Medicine, Medical Centre - Albert-Ludwig’s-University of Freiburg, Albert-Ludwig’s-University of Freiburg, Freiburg, Germany; 2 Department of Diagnostic and Interventional Radiology, Faculty of Medicine, Medical Centre - Albert-Ludwig’s-University of Freiburg, Albert- Ludwig’s-University of Freiburg, Freiburg, Germany; 3 Department of Anesthesiology and Critical Care, Faculty of Medicine, Medical Center - University of Freiburg, University of Freiburg, Freiburg I.Br, Freiburg, Germany; 4 Dep. Of Orthopedic Surgery, University Hospital Odense, Sdr. Boulevard 29, Odense C, 5000 Denmark

**Keywords:** Obesity, wounds and injuries, Venous thromboembolism, Tranexamic acid, resuscitation

## Abstract

**Purpose:**

Given obesity’s rising prevalence and its established role as an independent thromboembolic risk factor, potentially inducing a procoagulant state post-trauma, this study aimed to pinpoint key obesity-related factors influencing thromboembolic occurrences in this vulnerable patient group to guide interventions.

**Methods:**

This retrospective study analyzed a consecutive cohort of 1547 trauma patients age ≥ 18 years with an Injury-Severity-Score (ISS) ≥ 9 admitted to our level I trauma center between 01/2018 and 12/2024. Patients’ data were extracted from electronic medical records. Exclusions included pregnancy, malignant/neurodegenerative disease, prior thromboembolism, and inconclusive documentation. Risk factors and influencing factors regarding obesity to suffer post-traumatic thromboembolism were evaluated.

**Results:**

Older age, higher Body Mass Index (BMI), and greater Injury Severity Score (ISS) (*p* < 0.05 for all) were identified as significant independent predictors, with BMI revealing the strongest effect (OR 1.077, *p* = 0.001). In the obese cohort (BMI ≥ 30), administration rates of tranexamic acid (TXA) and erythrocytes did not significantly differ between the TE and non-TE-groups (*p* > 0.05). Hemoglobin levels were significantly lower in the TE group at 0, 24 and 48 h post trauma (*p* < 0.05), while International Normalized Ratio (INR) and Partial Thromboplastin Time (aPTT) did not significantly differ.

**Conclusion:**

Older patient age, higher BMI, and ISS are independent predictors of post-traumatic thromboembolism. Crucially, the administration of TXA and erythrocyte concentrates, essential for acute hemorrhage control, was not associated with an increased thromboembolic risk in the obese cohort. These findings support aggressive hemostatic resuscitation in high-risk obese patients.

## Introduction

Despite great improvements in patient trauma management in recent years, trauma remains a major clinical and socioeconomic burden, especially among the younger population. Disorders in energy utilization and metabolic dysregulation, including hyperglycemia and increased insulin resistance, are common in severely injured patients [1, 2]. Widely dispersed throughout the body, white adipose tissue is inherently associated with traumatic injury and is essential for maintaining energy homeostasis [3, 4]. However, adipose tissue is now understood to be itself a highly active endocrine organ that secretes a wide range of bioactive chemicals into the bloodstream, collectively referred to as adipokines, in addition to its conventional function as an energy reserve. These include classic hormones like leptin and adiponectin, growth factors, chemokines, cytokines, lipid mediators (e.g., prostaglandins), and various metabolites (e.g., fatty acids, nucleotides, nucleosides, lactate).

The global rise in prevalence of obesity is drawing greater attention to its health implications [5]. Obesity is acknowledged as an established independent and moderate risk factor for thromboembolic events [6–9]. Additionally, the risk in trauma patients to suffer such an event like pulmonary embolism or a venous thromboembolism (VTE) can be dangerously elevated and influenced by patient demographics, and prophylaxis type [10, 11]. Furthermore, evidence suggests that an elevated body mass index (BMI) and increased fat mass can shift the post-trauma coagulation state towards a procoagulant phenotype, thereby raising the risk of thrombosis and pulmonary embolism [7, 12]. The delicate balance between pro- and anti-inflammatory mediators as well as activators and coagulation inhibitors is a critical determinant in the management of severely injured patients [13, 14]. Detection of clinical thrombosis might be challenging as biomarkers like d-dimer levels are regularly elevated in trauma patients and clinical signs like swelling are masked in obese patients [15–17].

While adipose tissue serves as an energy store and organ-protecting cushion, the products it secretes are vital in regulating various physiological processes [4]. Recent meta-analyses indicate that patients with class 3 obesity face a higher risk of in-hospital mortality following trauma than individuals with a normal BMI [18]. Nevertheless, there are still too few thorough, extensive investigations needed to clarify these findings’ scientific significance. The interplay between obesity’s protective and detrimental factors in severely injured patients creates a complex balance that can be upset by various parameters [19, 20].

Furthermore, the potential for red blood cell (RBC) transfusions may heighten the risk of thromboembolic events—through mechanisms such as increased blood viscosity, oxidative stress, and inflammatory responses—necessitates careful assessment, especially in the already high-risk obese trauma patient population [21, 22]. Despite TXA’s proven efficacy in preventing or managing hemorrhage without raising the thrombosis risk [23–26], there’s persisting concern about its prothrombotic effects in major trauma patients already predisposed to thromboembolism due to obesity and injury severity [10, 27].

The aim of this study was to precisely define the major characteristics that influence obesity in relation to thromboembolic events in patients who have been seriously injured, evaluate their importance, and, as a result, assess potential therapeutic strategies. Our first hypothesis was that in severely injured patients, more obese patients carry a higher risk for suffering from thromboembolic events. The second hypothesis was that treatment with TXA as well as transfusion of red packed blood cells and platelet concentrates during trauma management also increase the risk for thromboembolic events.

## Materials and methods

The study was conducted in accordance with the Declaration of Helsinki (as revised in 2013). The protocol was reviewed and approved by the Institutional Review Board of the medical facility of the authors institution (approval No. 24–1512-S1-retro). Given the retrospective nature of this analysis of existing patient records, the requirement for individual patient informed consent was waived by the Ethics Committee. All patient data were anonymized and de-identified prior to analysis. This study followed the STROBE guidelines for observational studies (Strengthening the Reporting of Observational Studies in Epidemiology) and the RECORD guidelines (Reporting of studies Conducted using Observational Routinely collected Data) [28, 29].

We conducted a retrospective review on a consecutive cohort of patients admitted to authors institution between January 2018 and December 2024 after trauma. Patients were identified via a retrospective query of the hospital’s electronic medical records, in which severely injured trauma patients have been continuously prospectively registered with their respective ISS. Inclusion criteria were patient age ≥ 18 year and an ISS ≥ 9 that underwent a polytrauma-scan in the emergency department following traumatic injury. Patients’ characteristics, disease-specific information, laboratory parameters and radiologic characteristics were abstracted and transferred to an electronic spreadsheet. Exclusion criteria were pregnancy, malignant disease, life-shortening neurodegenerative disease as well as past thromboembolism in the patient’s history. Patient demographic data, including age, gender, height, weight, American Society of Anesthesiologists Physical Status Classification (ASA) and Body Mass Index (BMI), as well as their Injury Severity Score (ISS) after trauma, were documented. Additionally, the transfusion of red packed blood cells (RBC), platelet concentrates (PC), fresh frozen plasma (FFP) and the administration of tranexamic acid (TXA) (routinely not more than 2 g in total during first 24 h) at any given time during emergency and/or the stationary treatment were documented for each patient. Each patient’s premedication, specifically oral anticoagulation, was also documented. Laboratory parameters measured in the patients’ serum at the time of admission, 24 h, and 48 h post-trauma were also documented. Obesity was defined according to the World Health Organization (WHO) criteria as a Body Mass Index (BMI) of ≥ 30 kg/m².

The documented data was then correlated with a thromboembolic event, namely venous thromboembolism such as deep vein thrombosis (DVT), pulmonary embolism (PE) and arterial thromboembolism manifested as strokes, heart attacks, and peripheral arterial embolism.

We formed two groups to compare patients that suffered a thromboembolic event with those that did not:

1. Non-Thromboembolic event patients: “non-TE”
2. Thromboembolic event patients: “TE”

Laboratory parameters and emergency transfusions were correlated with thromboembolic events in obese patients (BMI ≥ 30) to evaluate their influence and predictive value for this special subset in trauma patients.

All patients were treated by specialized orthopedic trauma surgeons, specialized anesthesiologists and intensive care specialists. All patients received state of the art and guideline-compliant prophylaxis of thromboembolism with low-molecular-weight heparin, or in the case of therapeutic anticoagulation, according to the standard for achieving the corresponding therapeutic drug levels dependent on their underlying disease.

### Statistical analysis

All variables were evaluated for normality distribution using a combination of histograms, quantile–quantile (Q–Q) plots and Shapiro–Wilk tests. Descriptive statistics were summarized as means and standard deviations for quantitative variables and as counts and frequencies for categorical variables across all variables. Mann-Whitney-U test was chosen as a non-parametric test for comparison of groups. A multiple logistic regression was chosen to identify multiple independent variables. Statistical tests were calculated two-tailed using a 95% confidence level. Statistical significance was set at *p* < 0.05. 95% Confidence Intervals (95% CI) were reported where appropriate. Statistical analyses were performed with Graphpad Prism 10.6 (Graphpad, CA, San Diego).

## Results

### Sociodemographic data, BMI and ISS

**Fig. 1:**
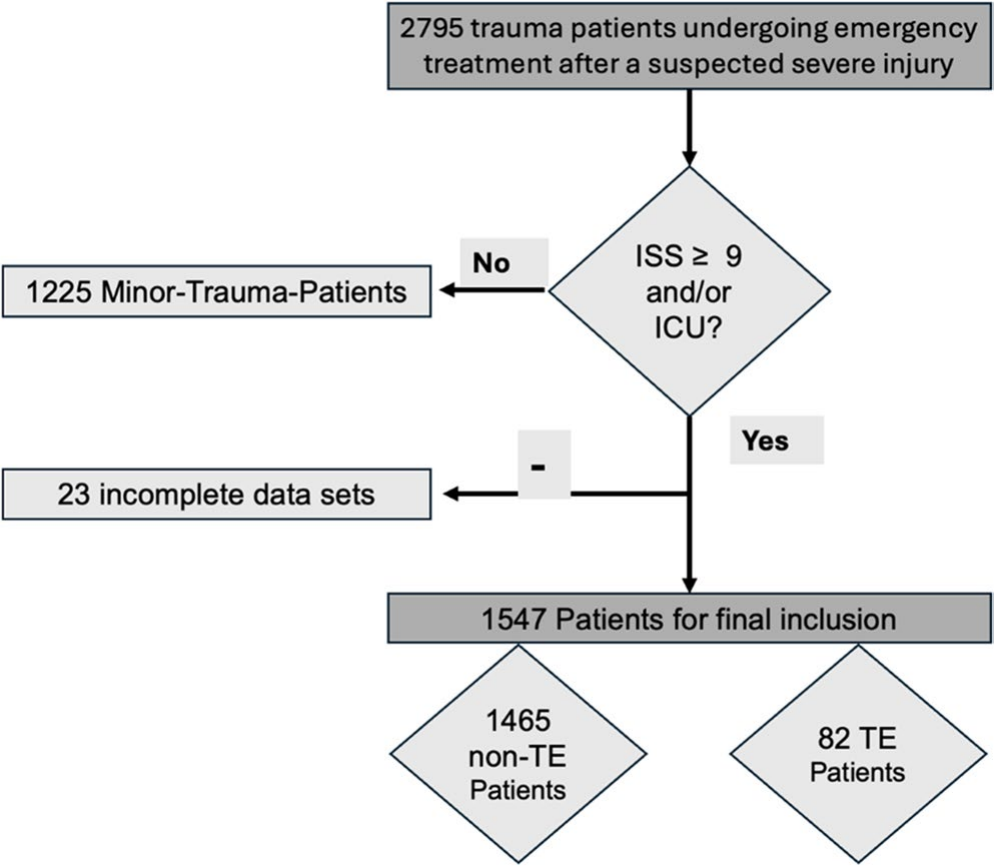
Patient flow chart illustrating flow for inclusion in our final data set of 1547 severely injured trauma patients (ISS > 9 and/or ICU treatment)

During January 2018 and December 2024, 1547 patients met our inclusion criteria, 82 of whom were TE patients and 1465 non-TE patients (Fig. 1). 24.6% were (*n* = 380) female and 75.4% (*n* = 1167) male patients. Mean age of the final data set was 52.0 ± 20.9 years (95%CI: 50.9–53.0, Range: 18–99). Mean BMI was 26.2 ± 4.6 (95%CI: 26.0–26.4, Range: 14.3–54.7) and mean ISS was 19.9 ± 11.4 (95%CI: 19.3–20.5, Range: 1–75).

### Identifying risk factors and influencing factors to suffer thromboembolism

Non-TE and TE groups were compared regarding possible influencing risk factors to suffer a thromboembolic event during inpatient treatment after suffering a severe injury (ISS ≥ 9) or intensive care treatment after injury (Table 1).

**Table 1:** Comparison of baseline characteristics between non-thromboembolic (non-TE) and thromboembolic (TE), presented as mean ± standard deviation, was well as their p value of difference

	Non-TE	TE	*p*-value
Patient age in years	51.6 ± 21.0	58.6 ± 19.6	0.005*
BMI	26.1 ± 4.5	28.0 ± 5.6	0.001*
ISS	19.6 ± 11.2	25.4 ± 13.4	< 0.001*
ASA	2.1 ± 0.9	2.2 ± 0.9	0.085

* Differences between groups were statistically significant

A multiple logistic regression analysis was then conducted to examine the relationship between a patient`s risk factors and their effects on the odds of thromboembolism individually. ASA prior to injury did not emerge as a significant predictor (*p* = 0.813). The ISS (*p* < 0.0001) and age (*p* = 0.0224) however were significant risk factors, while BMI was highly significant and even revealed the strongest effect among the predictors (*p* = 0.0011) (Table 2).

**Table 2:** Multiple logistic regression to identify independent risk factors for suffering a post-traumatic thromboembolic event

Variable	Odds Ratio (OR) Estimate (increase)	95% Confidence Interval (CI)	*p*-value
ASA prior to injury	0.9618	0.6954 to 1.327	0.813
ISS	1.037 (3.7%)	1.020 to 1.054	< 0.001*
BMI	1.077 (7.7%)	1.031 to 1.125	0.001*
Age	1.015 (1.5%)	1.002 to 1.029	0.022*

* Differences between groups were statistically significant

We also assessed oral anticoagulation and the effect of the transfusion of coagulation influencing drugs in our cohort’s 244 obese patients (BMI ≥ 30) upon admission or during their inpatient treatment in relation to suffering a thromboembolic. Patients not suffering a thromboembolic event (non-TE, *n* = 224) and those who did (TE, *n* = 20) were compared. This served to evaluate their individual influence on the risk to suffer a thromboembolic event in obese patients (Table 3.). In summary, we detected no statistically significant difference in the administration rates of the assessed blood products or oral anticoagulation between the obese patients (BMI ≥ 30) in our cohort who experienced a thromboembolic event and those who did not.

**Table 3:** Comparison of administration rates for medications and blood products in obese patients (BMI ≥ 30), categorized by thromboembolic event (TE versus non-TE). The data displays the percentage and number (n) of patients within each group

	Non-TE % (*n*)	TE % (*n*)	*p*-value
Oral anticoagulation	23.7 (53/224)	20.0 (4/20)	0.711
RBC	18.3 (41/224)	35.0 (7/20)	0.072
TXA	21.9 (49/224)	25.0 (5/20)	0.747
PC	8.5 (19/224)	20.0 (4/20)	0.091
FFP	13.0 (29/224)	15.0 (3/20)	0.794

Laboratory values were then analyzed at three time points (0 h, 24 h, and 48 h after the traumatic event) in obese patients (BMI ≥ 30; *n* = 244) to assess their predictive value regarding the occurrence of a thromboembolic event (TE-patients) versus no event (non-TE-patients) (Table 4.). Patients who suffered a thromboembolic event (TE) demonstrated significantly lower hemoglobin levels than those without an event (Non-TE) at all measured time points, while there were no statistically significant differences in the mean values for either INR or activated Partial Thromboplastin Time (aPTT) between the TE and Non-TE groups at any time point (0 h, 24 h, or 48 h).

**Table 4:** Laboratory values at 0 h, 24 h, and 48 h post-trauma in obese patients (BMI ≥ 30) to determine any association with the occurrence of a thromboembolic event. Data are presented as mean ± standard deviation

	Non-TE	TE	*p*-value
Hemoglobin 0	14.1 ± 2.3	12.3 ± 2.3	< 0.001*
INR 0	1.1 ± 0.3	1.1 ± 0.2	0.378
aPTT 0	28.4 ± 10.6	27.1 ± 7.4	0.464
Hemoglobin 24	11.4 ± 2.3	9.7 ± 1.9	0.004*
INR 24	1.2 ± 0.2	1.0 ± 0.0	0.645
aPTT 24	32.6 ± 11.7	32.1 ± 5.6	0.835
Hemoglobin 48	10.3 ± 2.3	8.7 ± 1.7	0.011*
INR 48	1.0 ± 0.2	1.0 ± 0.1	0.058
aPTT 48	33.1 ± 7.8	32.3 ± 3.4	0.968

* Differences between groups were statistically significant

## Discussion

The most important finding of this study is that obesity significantly increases a patient’s risk of developing procoagulant states after suffering a serious injury, which we have demonstrated on a large scale by evaluating 1547 severely injured trauma patients with an ISS ≥ 9 and/or who required intensive care unit therapy after trauma. BMI, ISS, and patient age emerged as statistically significant independent predictors, suggesting that not only more severe injuries and a higher age but also greater obesity independently raise the likelihood of developing thromboembolic complications.

Thromboembolic events and especially pulmonary embolism (PE) pose life-threatening complications during the post-trauma phase, once the severely injured patient has survived the initial acute phase [7, 10, 30]. The reported incidence of venous thromboembolism (VTE) in trauma patients varies widely, from 7% to 60%; it is influenced by patient demographics, detection methods, and prophylaxis type [30–32]. Without appropriate preventative measures, hospitalized major trauma patients face a significantly higher risk of developing VTE. This increased risk is classically attributed to Virchow’s Triad: endothelial injury, blood flow stasis, and intrinsic hypercoagulability [12, 33]. Beyond that, obesity significantly raises the likelihood to suffer thromboembolic conditions like VT and PE through a chronic inflammatory state, impaired fibrinolysis, and elevated levels of pro-clotting factors, all contributing to a hypercoagulable state [9, 34]. Data from this study confirms the assumption that in cases of major trauma, this inherent predisposition to clot formation is dramatically exacerbated in patients with high BMIs, making meticulous prophylactic strategies essential for this vulnerable population [34, 35].

Interestingly, in this study we found that the transfusion of erythrocyte concentrates, platelets, thrombocytes as well as TXA administration during both the emergency treatment and inpatient phase revealed no direct effect on the likelihood of suffering a thromboembolic event during stationary treatment. these common emergency interventions showed no impact on the likelihood to suffer thromboembolic events post-trauma or during the inpatient treatment phase in the obese patient specifically. This is a crucial finding, as especially TXA - a synthetic lysine analogue functioning by competitively inhibiting plasminogen throughout the body [36], and erythrocyte transfusions are vital interventions in acute trauma care controlling hemorrhage and maintaining oxygen delivery to significantly reduce mortality [37, 38]. About a third of bleeding trauma patients present indications of coagulopathy upon admission, while uncontrolled bleeding post-injury remains one of the primary causes of preventable deaths [26, 39]. Critically bleeding patients are more likely to develop multiple organ failure and face a higher mortality rate compared to those with similar injuries but without coagulopathy. Studies involving roughly one million patients have shown that TXA effectively prevents or manages hemorrhage without increasing the risk of thrombosis [23–25]. These studies mainly involved patients with perioperative or postpartum bleeding however, they did not evaluate severely injured obese trauma patients specifically. Concerns are growing regarding the potential prothrombotic effects of widespread TXA application especially in a patient population already predisposed to thromboembolism through their obesity and major injury severity [9, 10, 27]. RBC can also elevate the risk for thromboembolic events, particularly by increasing blood viscosity [40, 41], oxidative stress and hemolysis [42, 43] as well as an inflammatory response and platelet activation [44] - a mechanism whose impact has not been evaluated on the severely injured (in particular not the obese) trauma patient.

Our data suggests however, that both these life-saving measures can be safely administered in severely injured obese patients without directly raising their risk of thromboembolic events during their inpatient treatment. This facilitates decision making during emergency therapy, prioritizing hemorrhage control without undue concern for exacerbating the thrombotic risk from these interventions.

Our analysis of laboratory values from the obese (BMI ≥ 30) trauma patients only revealed a strong association between significantly lower Hb levels and the subsequent development of TE. Patients who suffered from TE consistently presented with lower mean Hb values at admission (0 h), 24 h, and 48 h post-trauma, suggesting that greater acute blood loss or early post-traumatic anemia may constitute a critical and somewhat neglected indicator of a higher risk for TE in this subset of obese and severely injured trauma patients. This finding is in line with literature linking hemorrhage and transfusion requirements to a pro-thrombotic state [45]. In important contrast to that, we noted that standardized coagulation markers such as INR and aPTT showed no significant difference between TE and non-TE patients at any measured time. This evidence indicates that the elevated risk for TE is probably not attributable to more serious abnormalities in measured intrinsic or extrinsic pathways, but rather to subtle changes such as fibrinolytic shutdown or platelet activation. Trauma specialists should therefore ensure heightened clinical vigilance and early treatment in obese trauma patients presenting with relevant anemia.

It is important to note, that our dependence on conventional coagulation assays such as INR and aPTT may obscure more nuanced hypercoagulable conditions. Viscoelastic assays (e.g., Rotational ThromboElastoMetry (ROTEM), Thromboelastography (TEG)) are progressively acknowledged in contemporary trauma management for their capacity to deliver an extensive, real-time evaluation of clot development, strength, and lysis [26]. Hypercoagulability associated with obesity is frequently identifiable solely by specialized assays, which demonstrate alterations in fibrinolytic shutdown and platelet activity that standard indicators do not capture [46]. The absence of a notable variation in INR and aPTT within our cohort may represent a restriction of the assay type, highlighting the necessity for further investigations employing viscoelastic testing in this particular patient population.

When it comes to the prevention and treatment of thromboembolic events, obese trauma patients pose a unique set of challenges, necessitating a sophisticated, individualized care strategy. Importantly, our study data demonstrate a clear link between a higher BMI and increased risk of thromboembolic events that must be acknowledged. To increase survival rates, outcomes, and quality of life, clinicians must carefully evaluate each obese trauma patient, considering a wide range of criteria. This includes a detailed inventory of the patient’s pre-existing illnesses and an examination of any medications the patient may be taking that may raise or lower their risk of clotting.

### Limitations

Since our findings relied on pre-existing clinical data that may include missing information, inconsistent data, or different collecting standards, this study has certain limitations. The first of these is its retrospective design. Screening for venous thromboembolism was not routinely performed. Exclusively thromboembolic events which became clinically apparent where counted. Second, our patient population may not accurately represent all obese patients with severe injuries, which could lead to selection bias. Confounding by indication is yet another drawback because treatments such as erythrocyte concentrates and tranexamic acid were given according to therapeutic necessity, which means that patients who received them might have been more ill and had an independent impact on our results. This study’s observational approach makes it impossible to demonstrate direct causation—only associations—which emphasizes the necessity of additional prospective research, ideally randomized controlled trials, to replicate our findings. Finally, the identification of significantly lower hemoglobin levels in individuals who experienced thromboembolic events introduces a crucial, unaccounted confounder: the timing of thromboprophylaxis commencement. Patients exhibiting initial or persistent severe hemorrhage, as seen by decreased hemoglobin levels, may have experienced a delayed commencement or temporary suspension of routine VTE prophylaxis due to clinician apprehensions about the danger of hemorrhage. The postponement of suitable prophylaxis is a significant clinical factor that may independently increase the risk of thromboembolism in this group. Due to the inconsistent availability of data on the precise timing of the initial prophylaxis medication for all patients, we were unable to account for this potential confounding variable related to prophylaxis scheduling. This must be recognized as a significant drawback of our observational strategy and is a crucial consideration for future prospective trials.

## Conclusions

Older patient age, higher BMI, and ISS are independent predictors of post-traumatic thromboembolism. Crucially, the administration of TXA and erythrocyte concentrates, essential for acute hemorrhage control, was not associated with an increased thromboembolic risk in the obese cohort. These findings support aggressive hemostatic resuscitation in high-risk obese patients.

## Data Availability

In accordance with ethical approval and patient consent, the data are considered confidential and are held securely by the authors’ institution. Data are available only upon reasonable request to the corresponding author, and only where that request aligns with institutional and national data sharing policies and is approved by the relevant Institutional Review Board (IRB)/Ethics Committee. Any data sharing would be subject to a formal data sharing agreement and may require data to be anonymized or aggregated to protect participant privacy.
